# Soil and water conservation practice effects on soil physicochemical properties and crop yield in Ethiopia: review and synthesis

**DOI:** 10.1186/s13717-022-00364-2

**Published:** 2022-02-07

**Authors:** Endale Bekele Jiru, Habtamu Temesgen Wegari

**Affiliations:** 1 College of Agriculture and Natural Resource, Dilla University, Dilla, Ethiopia; 2 College of Agriculture and Natural Resource, Bonga University, Bonga, Ethiopia

**Keywords:** Land degradation, Soil and water conservation, Crop yield, Soil physicochemical properties

## Abstract

**Background:**

Land degradation is an urgent agenda that requires great effort and resources to ameliorate. It worsens soil components through disrupting ecological functions and threatens agriculture production. To overcome it, different soil and water conservation (SWC) practices have been undertaken in numerous parts of Ethiopia. This paper aims to review the effects of SWC practices on soil physicochemical properties and crop yield. Data were collected from secondary sources via a computer library using various databases based on developed criteria. The collected data were organized, categorized, and analyzed through descriptive statistics. The mean difference of selected soil physicochemical properties obtained from treated and untreated farmland was tested using paired *t*-test. Factors influencing crop yield on treated farmland were determined by a multiple linear regression model.

**Results:**

SWC practices influenced the soil physicochemical properties and crop yield either positively or negatively. The mean values of available phosphorus (10.6 ppm, 8.7 ppm), total nitrogen (0.5%, 0.4%), soil pH (6.0%, 5.8%), soil organic matter (4.4%, 3.8%), and soil organic carbon (2.2%, 1.8%) were on treated and untreated farmland under physical SWC practices, respectively. Similarly, the mean values of these variables were higher on treated farmland than untreated farmland under both biological and integrated SWC practices. The mean value of bulk density was higher on untreated farmland than treated one and statistically significant under all SWC practices. Fanya juu and stone-faced soil bund constantly increased crop yield, whereas soil bund and stone bund did not.

**Conclusion:**

Proper implementation of SWC technologies through integrating physical and biological measures will boost the effectiveness of the practice in restoring soil physicochemical properties and improving crop yield. Meanwhile, government due attention paid for land resources management in Ethiopia, whereby the annual SWC and tree planting campaign underwent for a couple of decades, entails further scientific support for its efficacy.

## Introduction

Productive land is a cornerstone to global food security and environmental health, zero hunger, poverty eradication, and energy for all. It underpins the success of the entire 2030 Agenda for Sustainable Development (Desa 2016). Land degradation is recognized as a land productivity challenge and its severity is high in developing countries. Many scholars stated that it was aggravated by different driving forces that can potentially disrupt ecological processes. For example, the interconnecting between natural ecosystem and human being system is accounted as the main cause of land degradation (Hurni et al. 2010; Gashaw et al. 2014), such as population pressure (Pender and Gebremedhin 2004; Habtamu et al. 2014), land use/cover change (Emiru and Taye 2012; Meshesha et al. 2012), deforestation (Lambin et al. 2003; Gebru 2016), overgrazing (Kechero et al. 2013), improper land utilization and intensification of farming (Nyssen et al. 2015). Besides, a few natural driving forces like landslides (Broothaerts et al. 2012), and climate change (Deresa and Legesse 2015) are also included.

Soil erosion is a gradual process that occurs when the impact of water or wind detaches and removes soil particles, causing the soil to deteriorate and hence land to degrade (Hurni 1985; Hurni et al. 2010; Gashaw et al. 2014). Soil deterioration and low water quality due to erosion is a serious problem for agro-ecosystems productivity and water quality concerns. Controlling the sediment must be an integral part of any soil management system to improve water and soil quality, ecological functionality and hence enhancement of agro-ecosystems productivity. Although tolerable erosion is part of ecological process for the functionality and development of aquatic ecosystems, intolerable topsoil and agrochemicals transported by water into streams impact both productive agricultural land (on-site) and the water quality through sediment generation (off-site) (Boardman 2013). Therefore, the most effective way to minimize sediment production so as to stabilize ecological processes is the stabilization of the sediment source by controlling erosion (Demissie et al. 2013; Wolka et al. 2018).

In Ethiopia, soil erosion is severe mainly in the Highland part of the country. The mean annual soil loss was estimated to be 30.4 t/ha/year (Amsalu and Mengaw 2014), 23.7 t/ha/year (Gashaw et al. 2017), and 47.4 t/ha/year (Gelagay and Minale 2016). Besides, Teshome et al. (2013) also estimated to be 64.3 t/ha/year (in Debre Mewi) and 122.3 t/ha/year (in Anjeni) which in turn diminished crop yield and exposed the people to food insufficient. By these figures, soil losses were above the tolerable soil loss recommended for Ethiopia which ranges from 2 to 18 t/ha/year (Hurni 1985). As a consequence of land degradation, around 14 million hectares are severely eroded and a couple of million hectares reached an irreversible stage. Broadly, soil erosion is manifested itself in terms of onsite and offsite effects (Haile et al. 2006; Boardman 2013) affecting the ecological processes at large. For instance, soil organic matter and soil organic carbon loss by soil erosion in northern parts of the country (Amare et al. 2013; Adimassu et al. 2012) and caused topsoil quality and quantity deterioration. Subsequently, it restricted the ecological function and ecosystem services of soil as depletion of soil nutrients and soil fertility is impeding the productive capacity of the land. Furthermore, an offsite effect also caused physical damages and ecological disruptions through depositing soil constituents in irrigation schemes (Stroosnijder 2009), affect aquatic ecosystem health (Dersseh et al. 2019), and shortens the life span of hydraulic structures (Devi et al. 2008; Demissie et al. 2013; Wolka et al. 2018). Thus, soil erosion stimulated land degradation is directly deteriorating soil nutrients, soil fertility, and reduce crop yield. It inversely increases the cost to the farmers to redeposit a nutrient loss (Yirga and Hassan 2009).

In the last five decades, the Ethiopian Government paid due attention to land resources management; whereby the annual soil and water conservation and tree planting campaign for more than a decade is referred. Subsequently, the implemented technologies played a great role in ensuring soil nutrient and fertility in terms of reducing the run-off speed and breaking steepness, which in turn helps in reducing soil losses (Adimassu et al. 2012; Teshome et al. 2013; Mengistu et al. 2016), improving soil fertility (Belayneh et al. 2019; Tanto and Laekemariam 2019; Alemayehu et al. 2020), enhancing water-holding capacity of the soil, rehabilitating degraded land (Sinore et al. 2018; Terefe et al. 2020), and renovating land productiveness (Adgo et al. 2013; Ararso et al. 2016; Alemayehu et al. 2020). Besides, integrated physical and biological measures are reimbursed by way of modifying soil fertility and crop yield (Ayalew 2011), improving soil physicochemical properties (Terefe et al. 2020; Belayneh et al. 2019; Yakob et al. 2015), and built an opportunity for reusing of cultivated land which was preliminarily occupied by physical structures like planting fodder (Adimassu et al. 2017). Biological measures can speedily restore degraded land in terms of soil physical properties, improving soil fertility and nutrient status than physical structures (Terefe 2011). Furthermore, Tanto and Laekemariam (2019) also revealed that farmland treated by Fanya juu perceived more concentration of nutrients like availability of phosphorus (20.8 mg/kg), soil organic matter (2.78%), and soil organic carbon (1.61%) than the untreated one. Correspondingly, Sinore et al. (2018) observed that farmland treated by grass strip (elephant grass) exhibits a higher accumulation of total nitrogen and available phosphorus than untreated. Consequently, it improves the livelihood of the farmer through increasing crop yield (Amare et al. 2013; Waga and Jermias 2013). According to Waga and Jermias (2013), higher crop yield was computed from treated farmland (7.07 Q/ha) than untreated farmland (3.03 Q/ha). This does mean that 3.31 Q/ha crop yield was moreover obtained due to the intervention of SWC practices, which might help the farmers to maintain their food sufficiently.

Despite the advantages of SWC practices, there is also some physical structure that could not bring a positive result always on some specific output, particularly crop yield (Adimassu et al. 2012, 2017; Teshome et al. 2013). This was attributable to inappropriate construction of the physical structure (Demelash and Stahr 2010) and disconnection of physical with biological conservation measures (Sinore et al. 2018), which epitomize the land for further soil erosion and it reduces cultivable land and acts as a physical barrier (Wolka et al. 2018). Moreover, timely evaluation of the implemented SWC is overlooked in Ethiopia. This will probably deter the SWC practices. Many researchers had estimated the impacts of SWC practices on soil physicochemical properties and crop yield; however, their analysis does not illustrate consistency. As a result, just a few scholars’ evaluations had been solely centered on the effectiveness of SWC observed on chosen soil physicochemical properties (Sinore et al. 2018; Belayneh et al. 2019; Dagnachew et al. 2020). A few researchers have conducted on the effects of SWC practices on both soil physicochemical properties and crop yield (Tanto and Laekemariam 2019; Guadie et al. 2020). Some researchers have assessed the effects of SWC practice on its payback in terms of crop yield (Adgo et al. 2013; Teshome et al. 2013; Tesfaye et al. 2016). Generally, the evaluations conducted were inconsistent and overlooked the advantages of SWC technology in terms of time frame (yearly based) payback, and the inter-link between soil physicochemical properties and crop yield on treated farmland. Furthermore, they only concentrated on the effects of SWC practices directly without identifying the factors which maybe influence crop yield on treated farmland. Therefore, we compare the findings of various works, which portrayed the effects of SWC practices on the selected physicochemical properties and crop yield and then concoct it for policy formulation or modification. Considering that local and then regional processes triggered by SWC practices have a global impact, this review and synthesis result would help the scientific community as well. So, this paper aims to review and synthesize the effects of SWC practices on soil physicochemical properties and its reimbursements in the form of crop yield.

## Methods

### Data preparation

**Fig. 1:**
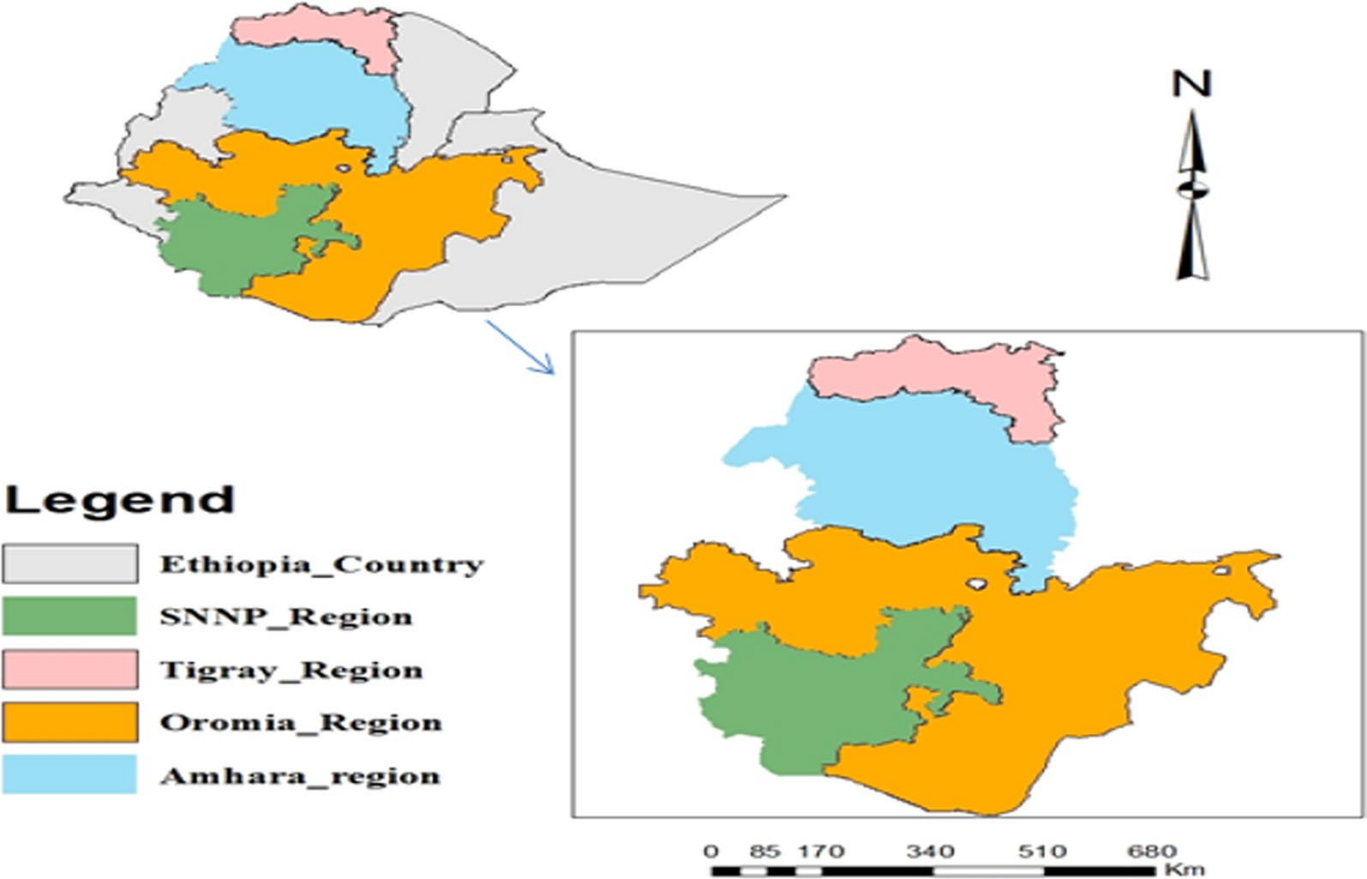
Map of the study area: SNNP_Region—refers to Southern Nations and Nationalities Peoples Region

To achieve the designed aim of this paper, data were collected from secondary sources via a computer library by using various databases: Web of Science (http://apps.webofknowledge.com), ResearchGate (https://www.researchgate.net), Google Scholar (scholar.google.com), and ScienceDirect (http://www.sciencedirect.com) based on developed criteria. These criteria are four regions of the study area where the major and target literature regarding physical, biological, and integrated SWC practices were sourced; year of publication, soil physicochemical properties, crop yield, and year (age) of the SWC practices. Detailed criteria and their categories (inclusion and exclusion) were depicted in the table and attached as supplementary data (Table S1). In general, around 31 kinds of literature published for researches conducted in the regions of the study area on the stated theme were included in this review from a proposed area (Fig. 1).

Land degradation mitigated through physical, biological, and agronomic SWC practices are common in Ethiopia. However, in this review, biological, physical, or integrated SWC practices were used and can be described as follows: (1) biological measure means a vegetative way of conserving soil and water which solely consists of grass strip or animal forage [e.g., elephant grass (*Pennisetum purpureum*), Sesbania (*Sesbania sesban*), Vetiver grass (*Vetiveria zizanioides*), tree lucerne (*Chamaecytisus palmensis*)], (2) whereas physical SWC practice consists of soil bund (graded or level), stone bund and Fanya juu (graded or level) and (3) the integrated SWC practice (selected physical structure stabilized with selected biological measure and with others). In some literature, SOM (soil organic matter) and SOC (soil organic carbon) were not given, so it was calculated by multiplying percent SOC by 1.724 (Jones 2001) and SOM by 0.58 (Guo and Gifford 2002), respectively. Most kinds of literature were excluded because they do not fulfill the designed criteria (Table 7).

### Data analysis

The collected data were organized, and categorized then inter into Microsoft Excel 2010 and copied into Statistical Package for Social Science (SPSS) Version 20 and ready for further synthesis. Accordingly, the effects of SWC practices on chosen soil physicochemical properties and crop yield were analyzed through descriptive statistics (e.g., mean, frequency, and percent). The mean difference of selected soil physicochemical properties obtained from treated and untreated farmland was tested statistically using paired *t*-test using SPSS. Factors influencing crop yield on treated farmland were determined by using a multiple linear regression model (Eq. 1). Finally, the consequence is introduced within the types of a table, and narrative:

1 $$Y_{i} = \beta 0 + \beta 1X1 + \beta 2X2 + \beta 3X3 + \beta 4X4 + \varepsilon_{i} ,$$

where *Y*_*i*_ is the *i*th amount of crop yield (Q/ha) obtained after the intervention of SWC practices, *β*0 is an intercept, *β*1 to *β*4 is the coefficient value of explanatory variables, *X*1 to *X*4 is an explanatory variable [year of SWC practices (year/age) (*X*1), mean annual temperature (°C) (*X*2), mean altitude (meter above sea level) (*X*3) and mean annual rainfall (mm) (*X*4)], which express *Y*_*i*_ and *ε*_*i*_ is an error term.

## Results

### Effects of SWC practices on selected soil physical property

#### Sand content

Table 1 shows the negative or positive effects of physical, biological, and integrated SWC practices on selected soil physical properties. Accordingly, approximately 17.86% (*N* = 28) and 7.14% (*N* = 28) of integrated SWC practices have positive and negative effects on sand content, respectively. Besides, physical and biological measures also imposed negative effects (19.36%, *N* = 93), and (30%, *N* = 10) on sand content, respectively. This means that most sand content is not changed due to the intervention of integrated SWC practices. Table 2 depicts the mean difference in the sand content of treated and untreated farmland. The mean difference value of sand content between treated and untreated farmland was estimated to be − 4.5 (− 16.4%) and − 1.5 (− 3.68%) by biological measure and physical structures, respectively, and statistically insignificant.

**Table 1:** Effects of SWC practices on soil physical properties as reported in reviewed articles

Types of SWC practices	Parameters	Observed numbers (*N*)	Effects	%	Author(s)
Physical (*N* = 93)					
Fanya juu	BD (g/cm)	5	−	5.38	Hailu et al. (2012), Dagnachew et al. (2020)
Soil bund		8	−	8.60	Degu et al. (2019), Mohammed et al. (2020), Tadesse et al. (2016), Guadie et al. (2020), Husen et al. (2017), Bezabih et al. (2016)
Stone bund		3	−	3.23	Hishe et al. (2017), Demelash and Stahr (2010)
		2	+	2.15	Mesfin et al. (2018), Belayneh et al. (2019)
Stone-faced soil bund		3	−	3.23	Hailu (2017), Guadie et al. (2020)
Fanya juu	Clay (%) 13.98 (+) 11.83 (−)	2	+	2.15	Tanto and Laekemariam (2019), Hailu et al. (2012)
		2	−	2.15	
Stone-faced soil bund		1	+	1.08	Guadie et al. (2020)
		2	−	2.15	Hailu (2017)
Stone bund		4	+	4.30	Wolka et al. (2011), Mesfin et al. (2018), Hishe et al. (2017)
		2	−	2.15	Wolka et al. (2011), Demelash and Stahr (2010)
Soil bund		6	+	6.45	Guadie et al. (2020), Degu et al. (2019), Wolka et al. (2011), Belayneh et al. (2019), Bezabih et al. (2016), Terefe et al. (2020)
		5	−	5.38	Wolka et al. (2011), Mohammed et al. (2020), Tadesse et al. (2016), Amdemariam et al. (2011)
Fanya juu	Silt (%) 12.91 (+) 12.91(−) 0	4	−	4.30	Tanto and Laekemariam (2019), Hailu et al. (2012), Dagnachew et al. (2020)
		1	+	1.08	Tanto and Laekemariam (2019)
Stone-faced soil bund		1	+	1.08	Guadie et al. (2020)
		1	−	1.08	Hailu (2017)
Stone bund		4	+	4.30	Wolka et al. (2011), Hishe et al. (2017), Mesfin et al. (2018)
		2	−	2.15	Wolka et al. (2011), Demelash and Stahr (2010)
Soil bund		5	−	5.38	Wolka et al. (2011), Bezabih et al. (2016), Terefe et al. (2020), Belayneh et al. (2019)
		6	+	6.45	Wolka et al. (2011), Guadie et al. (2020), Degu et al. (2019), Tadesse et al. (2016), Mohammed et al. (2020), Amdemariam et al. (2011)
Fanya juu	Sand (%) 19.36 (−) 6.45 (+)	2	+	2.15	Tanto and Laekemariam (2019), Dagnachew et al. (2020)
		1	−	1.08	Tanto and Laekemariam (2019)
Stone-faced soil bund		3	−	3.23	Guadie et al. (2020), Hailu et al. (2012)
Stone bund		4	−	4.30	Wolka et al. (2011), Mesfin et al. (2018), Hishe et al. (2017)
		2	+	2.15	Wolka et al. (2011), Demelash and Stahr (2010)
Soil bund		2	+	2.15	Amdemariam et al. (2011), Wolka et al. (2011)
		10	−	10.75	Wolka et al. (2011), Guadie et al. (2020), Degu et al. (2019), Tadesse et al. (2016), Mohammed et al. (2020), Bezabih et al. (2016), Terefe et al. (2020), Belayneh et al. (2019), Mesfin et al. (2018)
Biological (*N* = 10)	BD (g/cm^3^)	3	−	30	Sinore et al. (2018), Gesesse et al. (2013)
	Clay (%)	2	+	20	Sinore et al. (2018)
	Silt (%)	2	−	20	Sinore et al. (2018)
	Sand (%)	3	−	30	Sinore et al. (2018), Gesesse et al. (2013)
Integrated (*N* = 28)	BD (g/cm^3^)	7	−	25.00	Tanto and Laekemariam (2019), Tadesse et al. (2016), Amdemariam et al. (2011), Demelash and Stahr (2010)
	Clay (%)	5	−	17.86	Tanto and Laekemariam (2019), Amdemariam et al. (2011)
		2	+	7.14	Tadesse et al. (2016), Demelash and Stahr (2010)
	Silt (%)	5	+	17.86	Tanto and Laekemariam (2019), Tadesse et al. (2016);
		2	−	7.14	Demelash and Stahr (2010), Amdemariam et al. (2011)
	Sand (%)	2	−	7.14	Tanto and Laekemariam (2019), Tadesse et al. (2016)
		5	+	17.86	Demelash and Stahr (2010), Amdemariam et al. (2011)

(+) indicates the parameters improved or changed; (−) indicates the parameters not improved or not changed, and (0) indicates zero effects. This table simply depicted the analyzed results obtained from selected articles

**Table 2:** Effects of SWC practices on the mean value of soil physical properties in the study area

Parameter	Treated	Untreated	Change	Change %	*P*-value	Author(s)
Physical						
BD (g/cm^3^)	1.18 (22)	1.25 (22)	− 0.06	− 5.21	0.00**	Amdemariam et al. (2011), Hailu et al. (2012), Hailu (2017), Hishe et al. (2017), Tanto and Laekemariam (2019), Belayneh et al. (2019), Degu et al. (2019), Guadie et al. (2020), Mohammed et al. (2020), Tadesse et al. (2016), Demelash and Stahr (2010), Dagnachew et al. (2020), Bezabih et al. (2016), Husen et al. (2017), Terefe et al. (2020), Mesfin et al. (2018), Selassie et al. (2015)
Silt (%)	27.91 (25)	26.32 (25)	1.59	6.05	0.12	
Sand (%)	37.05 (25)	38.47 (25)	− 1.47	− 3.68	0.31	
Clay (%)	34.91 (24)	35.16 (24)	− 0.25	− 0.72	0.89	
Biological						
BD (g/cm^3^)	1.16 (3)	1.30 (3)	− 0.14	− 10.74	0.03*	Sinore et al. (2018), Gesesse et al. (2013)
Silt	29.00 (2)	41.00 (2)	− 12.00	− 29.27	0.03*	
Sand	23.00 (2)	27.50 (2)	− 4.50	− 16.36	0.07	
Clay	48.00 (2)	31.50 (2)	16.50	52.38	0.04*	
Integrated SWC practices						
BD (g/cm^3^)	1.17 (7)	1.27 (7)	− 0.10	− 7.78	0.00**	Amdemariam et al. (2011), Tanto and Laekemariam (2019), Tadesse et al. (2016), Demelash and Stahr (2010)
Silt (%)	30.29 (7)	26.72 (7)	3.57	13.35	0.19	
Sand (%)	26.34 (7)	20.58 (7)	5.76	28.00	0.11	
Clay (%)	43.36 7)	52.68 (7)	− 9.32	− 17.69	0.06	

**, *Indicates the statistical significance at 1% and 5%, respectively

#### Silt content

Table 1 reports on the effects of SWC practices on silt soil property where by integrated approach (+ 17.76%, *N* = 28) was modified the silt content, whereas biological measure was not changing (− 20%, *N* = 10), and that of physical structures (+ 12.91%, *N* = 93) have zero effect on silt content of treated farmland. Similarly, the mean difference value of silt content was relatively expressed (Table 2). The mean difference value of silt content between treated and untreated farmland was estimated to be 1.59 (6.05%), and 3.55 (13.35%) under physical, and integrated, respectively. It is not statistically significant among treated and untreated farmlands. This shows that the removability of silt texture and its deposition are relatively equal.

#### Clay content

Table 1 reports the effects of physical SWC practices on clay property. The reviewed articles indicated that clay soil content was not significantly improved by SWC practices. It was positively affected by physical structure (13.98%, *N* = 93) and biological measures (20%, *N* = 10) while negatively affected by integrated SWC (− 17.86%, *N* = 28). The mean difference value of clay content between treated and untreated farmland was 16.5 (52.4%) under biological measure and statistically significant (*p* < 0.05) (Table 2).

#### Bulk density

Generally bulk density is negatively influenced by soil and water conservation practices (Table 1). This implies that it was not improved under all SWC practices except under a few physical structures (e.g., stone bund) (2.15%, *N* = 93). In other words, SWC practices did not affect soil bulk density. The mean difference value of bulk density decreased by − 0.14 (− 10.74%), − 0.10 (7.78%), and − 0.06 (5.21%) on farmland treated by biological, integrated, and physical SWC practices, respectively (Table 2).

### Effects of SWC practices on soil chemical property

#### Soil pH

Soil reaction (pH) is the indicator of soil acidity or alkalinity of soil chemical properties. According to this assessment, soil pH value was either positively or negatively affected by SWC practices (Table 3). As reviewed papers indicated, physical structure (11.77%, *N* = 136), biological measure (15%, *N* = 20), and integrated measures (9.38%, *N* = 32) positively affected soil pH value. This implies that there is an improvement in pH values due to SWC intervention. The mean difference value of pH was estimated to be 0.22 (3.84%), 0.42 (7.82%), and 0.36 (6.06%) on farmland treated by physical, biological, and integrated SWC practices, respectively (Table 4). A higher pH value was recorded in treated farmland than the untreated one suggesting that soil acidity is relatively improved (reduced).

**Table 3:** Effects ofSWC practices on selected soil chemical properties as reported in reviewed articles

Parameter	*N*	Effects^a^	%	Author(s)
Physical (*N* = 136)				
pH				
Fanya juu	3	+	2.21	Tanto and Laekemariam (2019), Hailu et al. (2012)
	2	−	1.47	Hailu et al. (2012), Amare et al. (2013)
Stone-faced soil bund	4	+	2.94	Hailu (2017), Guadie et al. (2020), Asnake and Elias (2019)
Stone bund	3	+	2.21	Alemayehu et al. (2020), Hishe et al. (2017), Demelash and Stahr (2010)
Soil bund	6	+	4.41	Guadie et al. (2020), Degu et al. (2019), Belayneh et al. (2019), Mohammed et al. (2020), Husen et al. (2017), Demelash and Stahr (2010)
	2	−	1.47	Bezabih et al. (2016), Terefe et al. (2020)
SOC (%)				
Fanya juu	6	+	4.41	Hailu et al. (2012), Amare et al. (2013), Dagnachew et al. (2020)
Soil bund	11	+	8.09	Adimassu et al. (2012), Tanto and Laekemariam (2019), Guadie et al. (2020), Degu et al. (2019), Belayneh et al. (2019), Mohammed et al. (2020), Tadesse et al. (2016), Husen et al. (2017), Bezabih et al. (2016), Terefe et al. (2020)
	1	0	0.74	Selassie et al. (2015)
Stone bund	1	−	0.74	Wolka et al. (2011)
	7	+	5.15	Alemayehu et al. (2020), Hishe et al. (2017), Mesfin et al. (2018)
Stone-faced soil bund	4	+	2.94	Hailu (2017), Guadie et al. (2020), Asnake and Elias (2019)
SOM (%)				
Fanya juu	6	+	4.41	Hailu et al. (2012), Tanto and Laekemariam (2019), Dagnachew et al. (2020)
Stone-faced soil bund	4	+	2.94	Hailu (2017), Guadie et al. (2020), Asnake and Elias (2019)
Stone bund	7	+	5.15	Alemayehu et al. (2020), Wolka et al. (2011), Hishe et al. (2017), Mesfin et al. (2018), Demelash and Stahr (2010)
	1	−	0.74	Wolka et al. (2011)
Soil bund	11	+	8.09	Wolka et al. (2011), Adimassu et al. (2012), Guadie et al. (2020), Degu et al. (2019), Belayneh et al. (2019), Mohammed et al. (2020), Terefe et al. (2020)
	1	−	0.74	Wolka et al. (2011)
Ava.P (Pmm)				
Fanya juu	4	+	2.94	Hailu et al. (2012), Tanto and Laekemariam (2019), Amare et al. (2013)
Stone-faced soil bund	3	+	2.21	Hailu (2017), Guadie et al. (2020), Asnake and Elias (2019)
Stone bund	2	+	1.47	Alemayehu et al. (2020), Hishe et al. (2017)
	3	−	2.21	Wolka et al. (2011)
Soil bund	10	+	7.35	Wolka et al. (2011), Adimassu et al. (2012), Guadie et al. (2020), Degu et al. (2019), Belayneh et al. (2019), Mohammed et al. (2020), Tadesse et al. (2016), Husen et al. (2017), Bezabih et al. (2016), Terefe et al. (2020)
	4	−	2.94	Wolka et al. (2011), Adimassu et al. (2012), Belayneh et al. (2019)
TN (%)				
Fanya juu	4	+	2.94	Hailu et al. (2012), Amare et al. (2013), Dagnachew et al. (2020)
Stone-faced soil bund	4	+	2.94	Hailu (2017), Guadie et al. (2020), Asnake and Elias (2019)
Stone bund	6	+	4.41	Demelash and Stahr (2010), Wolka et al. (2011), Hishe et al. (2017)
Soil bund	4	−	2.94	Mohammed et al. (2020), Bezabih et al. (2016), Adimassu et al. (2012)
	11	+	8.09	Wolka et al. (2011), Amdemariam et al. (2011), Adimassu et al. (2012), Guadie et al. (2020), Degu et al. (2019), Belayneh et al. (2019), Terefe et al. (2020)
Biological (*N* = 20)				
pH	3	+	15	Sinore et al. (2018), Hailu et al. (2020)
SOC (%)	4	+	20	
SOM (%)	4	+	20	Sinore et al. (2018), Gesesse et al. (2013)
TN (%)	1	−	5	Hailu et al. (2020)
	4	+	20	Sinore et al. (2018), Hailu et al. (2020), Gesesse et al. (2013)
Ava.P (ppm)	4	+	20	
Integrated (*N* = 32)				
pH	3	+	9.38	Tanto and Laekemariam (2019), Tadesse et al. (2016)
	2	−	6.25	Demelash and Stahr (2010), Yakob et al. (2015)
SOC (%)	8	+	25.00	Tanto and Laekemariam (2019), Tadesse et al. (2016), Demelash and Stahr (2010)
SOM (%)	8	+	25.00	
Ava.P (ppm)	4	+	12.50	
	1	−	3.13	Yakob et al. (2015)
TN (%)	6	+	18.75	Demelash and Stahr (2010), Yakob et al. (2015), Amdemariam et al. (2011)

^a^(+) indicates the parameters improved or changed and (−) indicates the parameters not improved or not changed, and (0) indicates zero effects

This table simply depicted the analyzed result obtained from selected articles

**Table 4:** SWC practices effects on the mean value of soil chemical properties in the study area

Parameters	Treated	Untreated	Change	Change %	*P*-value	Author(s)
Physical						
pH	6.02 (23)	5.79 (23)	0.22	3.84	0.01*	Wolka et al. (2011), Amdemariam et al. (2011), Hailu et al. (2012), Hailu (2017), Hishe et al. (2017), Tanto and Laekemariam (2019), Belayneh et al. (2019), Degu et al. (2019), Guadie et al. (2020), Mohammed et al. (2020), Tadesse et al. (2016), Demelash and Stahr (2010), Dagnachew et al. (2020), Bezabih et al. (2016), Husen et al. (2017), Terefe et al. (2020); Mesfin et al. (2018), Amare et al. (2013), Asnake and Elias (2019), Alemayehu et al. (2020), Adimassu et al. (2012)
TN (%)	0.45 (31)	0.4 (31)	0.054	13.35	0.00**	
Ava.P (ppm)	10.62 (31)	8.65 (31)	1.97	22.78	0.087	
SOM (%)	4.4 (35)	3.75 (35)	0.65	17.34	0.00**	
SOC (%)	2.17 (35)	1.79 (35)	0.39	21.67	0.00**	
Biological						
pH	5.79 (3)	5.37 (3)	0.42	7.82	0.09	Hailu et al. (2020), Sinore et al. (2018)
TN (%)	0.21 (4)	0.18 (4)	0.03	18.75	0.18	Hailu et al. (2020), Sinore et al. (2018, Gesesse et al. (2013)
Ava. P (Ppm)	11.39 (4)	7.49 (4)	3.91	52.20	0.22	
SOM (%)	3.73 (4)	2.46 (4)	1.27	51.76	0.08	Hailu et al. (2020), Sinore et al. (2018), Gesesse et al. (2013)
SOC (%)	2.16 (4)	1.42 (4)	0.74	51.76	0.08	
Integrated						
pH	6.33 (5)	5.97 (5)	0.36	6.06	0.04*	Tanto and Laekemariam (2019), Tadesse et al. (2016), Demelash and Stahr (2010), Yakob et al. (2015)
TN (%)	0.22 (6)	0.20 (6)	0.08	6.75	0.01*	Amdemariam et al. (2011), Tadesse et al. (2016), Demelash and Stahr (2010), Yakob et al. (2015)
Ava. P (Ppm)	15.29 (5)	8.23 (5)	7.06	85.83	0.08	Tanto and Laekemariam (2019), Tadesse et al. (2016), Demelash and Stahr (2010), Yakob et al. (2015)
SOM (%)	3.42 (8)	1.88 (8)	1.54	82.12	0.01*	Amdemariam et al. (2011), Tanto and Laekemariam (2019), Tadesse et al. (2016), Demelash and Stahr (2010), Yakob et al. (2015)
SOC (%)	1.98 (8)	1.09 (8)	0.90	82.35	0.01*	

**, *Indicate the statistical significance at 1% and 5%, respectively

#### Soil organic carbon

SOC concentration was affected by SWC practices (Table 3). Accordingly, biological measures (20%, *N* = 20) and integrated (25%, *N* = 32) and physical structure (20%, *N* = 136) positively affected soil organic carbon. The analysis result shows the mean difference value of SOC was estimated to be 0.39 (21.67%), 0.74 (51.76%), and 0.9 (82.25%) on treated farmland under physical, biological, and integrated SWC practices, respectively (Table 4).

#### Total nitrogen (TN)

As revealed in the review result the concentration of total nitrogen is either positively or negatively affected by the physical soil and water conservation practices. Consequently, biological (20%, *N* = 20) and physical structure (18.38%, *N* = 136) measures modified the total nitrogen accumulation on farmland (Table 3). In contrast, it was also negatively affected by biological (5%, *N* = 20) and physical (2.94%, *N* = 136) SWC practices. The mean difference value of TN was 0.054 (13.35%), 0.03 (18.75%), and 0.08 (6.75%) between treated and untreated farmland under physical, biological, and integrated SWC practices, respectively (Table 4). It reflected a positivity of SWC practice on TN of soils in the study area.

#### Available phosphorus

The intervention of SWC practices also affected soil phosphorus availability. It was improved by physical structure (17.77%, *N* = 136), integrated (12.5%, *N* = 32), and biological measure (4%, *N* = 20) (Table 3). According to many scholars' opinions, the availability of phosphorus varied among conserved and non-conserved farmland. The mean difference value of available phosphorus was 1.97 (22.78%), 3.91 (52.2%), and 7.06 (85.83%) between treated and untreated farmland under physical, biological, and integrated SWC practices, respectively (Table 4).

#### Soil organic matter (SOM)

Soil and water conservation practices positively influenced SOM (Table 4). The mean value of SOM was (4.4 pmm, 3.75 ppm), (3.73 ppm, 2.46 ppm) and (3.42 ppm, 1.88 ppm) (Table 5) with its mean difference of 0.65%, 1.27%, and 1.54% on treated and untreated farmland under physical, biological, and integrated SWC practices, respectively.

### Effects of SWC practices on crop yield

Table 5 describes the effects of soil and water conservation practices on crop yield. The result shows land treated by Fanya juu increases crop yield minimum by 8.1% at 2 years and maximum by 204% at 25 years when compared with untreated farmland. Soil bund comparatively increases crop yield minimum by 46.7% at nine ages and a maximum of 87.7% at twelve ages. Besides, the stone-faced soil bund can increase crop yield up to 49.6% at 10 years old. Furthermore, stone bund minimum increases crop yields up to 4.14% at 3 years and a maximum up to 9.46% at 5 years old. This suggested that most physical structure increases crop yield if it is properly constructed and managed following the soil and water conservation guideline. Integration of Fanya juu with various grasses (e.g., elephant grass, S*esbania*, desho grass, and pigeon pea) relatively increases crop yield minimum up to 9.31% and maximum up to 72.9%. Besides, soil bund integrated with tree lucerne can increase crop yield up to 56.63%. From previously argued, integrated SWC practice has a significant impact on soil property, reducing soil erosion, and increasing the water-holding capacity of the soil, which in turn increases land productivity.

**Table 5:** Effects of SWC practices on the mean of crop yield (Q/ha where IQ = 100 kg/ha) as reported in reviewed articles

Types of SWC practices	Types of crop	Treated (Q/ha)	untreated (Q/ha)	Change	Changes (%)	Author(s)
Physical						
Fanya juu—5 yrs	Wheat	33.30	24.70	8.6	34.82	Tanto and Laekemariam (2019)
Fanya juu—2 yrs	Wheat	26.70	24.70	2	8.10	
Fanya juu—25 yrs	Wheat	10.77	6.56	4.21	64.16	Amare et al. (2013)
Fanya juu—25 yrs	Maize	26.95	10.73	16.22	151.20	
Fanya juu—25 yrs	Barley	18.60	6.10	12.5	204.92	Adgo et al. (2013)
Fanya juu—25 yrs	Teff	9.50	4.90	4.6	93.88	
Fanya juu—25 yrs	Maize	17.30	7.70	9.6	124.68	
Soil bund—10 yrs	Barley	22.22	15.10	7.12	47.15	Guadie et al. (2020)
Soil bund—3 yrs	Barley	26.70	28.70	− 2	− 6.97	Adimassu et al. (2012)
Soil bund—4 yrs	Barley	26.30	28.20	− 1.9	− 6.74	
Soil bund—5 yrs	Barley	30.40	32.80	− 2.4	− 7.32	
Soil bund—9 yrs	Barley	17.13	11.66	5.47	46.87	Amdemariam et al. (2011)
Stone-faced soil bund—10 yrs	Barley	22.59	15.10	7.49	49.60	Guadie et al. (2020)
Stone bund—3 yrs	Teff	7.35	6.18	1.17	18.93	Teshome et al. (2013)
Stone bund—3 yrs	Wheat	7.19	6.25	0.94	15.04	
Stone bund—3 yrs	Barley	9.80	9.41	0.39	4.14	
Stone bund—3 yrs	Maize	16.67	13.34	3.33	24.96	
Stone bunds—5 yrs	Sorghum	20.59	18.81	1.78	9.46	Alemayehu et al. (2020)
Stone bunds—5 yrs	Chickpea	14.41	9.64	4.77	49.55	
Stone bund—3 yrs	Finger millet	14.71	14.92	− 0.21	− 1.41	Teshome et al. (2013)
Integrated						
Fanya juu + grasses^a^—2 yrs	Wheat	27.00	24.70	2.3	9.31	Tanto and Laekemariam (2019)
Fanya juu + grasses^a^—5 yrs	Wheat	42.70	24.70	18	72.87	
Soil bund + tree Lucerne—6 yrs	Barley	12.84	9.44	3.4	36.01	Amdemariam et al. (2011)
Soil bund + tree Lucerne—9 yrs	Barley	18.79	12.00	6.79	56.63	
Soil bund + vetiver—9 yrs	Barley	11.88	9.49	2.39	25.10	

^a^Either elephant, Sesbania, Desho or pigeon pea

### Factors affecting crop yield on treated farmland

Multiple linear regression model analysis shows that all the four variables considered significantly affected crop yield obtained from treated farmland (*p* < 0.05 and < 0.01). Accordingly, crop yield was positively influenced by the year (age) of soil and water conservation practices and mean annual rainfall and negatively influenced by mean annual temperature and altitude. The multiple coefficients of determination, *R*^2^ was above the moderate level of fitness, which showed that 86.1% of the variation of crop yield could be explained by the regression (Table 6).

**Table 6:** Multiple linear regression results of reviewed articles

Determinant factors	Unstandardized coefficients		Standardized coefficients	*T*	Sig.	Collinearity statistics	
(Constant)	*B*	Std. error	Beta			Tolerance	VIF
Year of SWC (age)	46.66	9.40		4.96	0.00**		
Mean annual rainfall	2.91	0.74	0.98	3.96	0.00**	0.23	4.43
Mean attitude	− 0.01	0.00	− 0.31	− 2.37	0.04*	0.80	1.25
Mean annual temperature	− 0.01	0.01	− 0.88	− 4.59	0.00**	0.38	2.66
*R*^2^ = 86.1	Adjusted *R*^2^ = 80.5			*F* = 15.4, *P* = 0.00			

*VIF* variance inflation fact

**, *Statistically significant at 1% and 5%

## Discussion

### Effects of SWC practices on physicochemical properties

The intervention of SWC practices had either a substantial or insignificant impact on soil physicochemical properties. Treated farmland has a significantly lower mean value of sand content as compared with untreated farmland. This suggests relative effects of SWC practice against soil erosion, which in turn increase the deposition of soil particles either by trapping the soil particle comedown or embracing on the field. Notably, fine particles like clay and silt textures have easily been taken away by transportation and translocation of run-off and soil erosion from untreated farmland. As a result, the concentration of sand content is relatively higher in untreated land-use types. This agrees with Hishe et al. (2017) and Belayneh et al. (2019), who confirmed that treated farmland has a lower mean value of sand content than the untreated one.

Silt content is relatively improved under farmland treated by the combination of the physical structure with various grasses or animal forage and physical structure than biological measure. The highest mean value of silt content was computed from treated farmland than the untreated one. This finding is consistent with the findings of Belayneh et al. (2019) and Bezabih et al. (2016), who reported that silt fraction is relatively higher on conserved than non-conserved land. However, sometimes the mean values of sand content and silt are beyond the effect of SWC practices. This is directly connected with soil parent materials rather than hosted practices. For example, sand content is high on treated farmland. This agrees with Dagnachew et al. (2020), who reported that mean sand content is higher on treated land than untreated land in Geshy micro-catchment, Gojeb River, Ethiopia. Similarly, silt fraction is higher on untreated farmland than treated one − 12.0 (− 29.3%) under biological measure and statistically significant (*p* < 0.05). This agrees with Dagnachew et al. (2020), who confirmed silt content can be higher on non-conserved land than conserved one. Besides, Mengistu et al. (2016) and Demelash and Stahr (2010) also reported similar results. This is directly connected with the preliminary story of soil’s parent materials from the soils that were formed, which has a basaltic trap series of volcanic eruptions. This kind of result occurred from the feature of the study area.

The difference of mean value of clay was significant (*p* < 0.05) among treated and untreated farmland under the biological intervention measure. The presence of vegetative cover plays an incredible role in two ways: delaying the movement of clay particles and improving organic matters through decay. Similarly, Hishe et al. (2017) reported that vegetative availability can limit the movement of clay particles by water or wind erosion. Sinore et al. (2018) also revealed that farmland treated with biological measures (e.g., elephant grass and sesbania) has higher clay soil content than untreated. However, its mean value difference was negative, − 0.25 (− 0.72%) and − 9.32 (− 17.69%), which was not significantly changed on farmland treated by physical structure (*p* < 0.05) and integrated SWC practice, respectively. This disagrees with findings reported by Wolka et al. (2011), Adimassu et al. (2012), Belayneh et al. (2019), and Dagnachew et al. (2020), who found that soil clay proportion is higher on conserved land than no-conserved land. But, it agrees with Demelash and Stahr (2010), who reported that a higher mean value of clay was registered on non-conserved than conserved land. This kind of outcome irregularly happened when the soil was exposed to soil erosion due to tillage practice and the surplus water brought the subsoil to topsoil which is naturally richest in clay contents. Notably, fine particles are not easily taken away through transportation and translocation of run-off and soil erosion. As a result, the concentration of clay texture is relatively plentiful on the conserved land. This shows SWC practices contribute substantial roles against soil erosion, reduce the removal of crop residues, soil loss, and improve soil fertility. The mean value of bulk density was an additional indicator in which it was very strong statistically significant between treated and untreated farmland at *p* ≤ 0.01. This implies that untreated farmland has a higher bulk density than treated one. A similar finding was reported from different places. For example, Sinore et al. (2018) from Lemo District of Southern Ethiopia, reported that the mean value of soil bulk density lower under land covered with elephant grass (1.12 g/cm^3^) and Sesbania (1.08 g/cm^3^) but higher (BD = 1.26 g/cm^3^) on degraded grazing land. They also further discussed that vegetation or plant species can restore soil organic matter, reduce soil erosion, decrease soil bulk density and increase soil porosity, which is inversely on bare land. Similarly, Demelash and Stahr (2010) from South Gonder, North-Western Highlands of Ethiopia, show a significant difference of bulk density between conserved and un-conserved land. Besides, Hishe et al. (2017) also reported that bulk density is high on untreated farmland due to the presence of sand content from Middle Silluh Valley, northern Ethiopia. Untreated cultivated land is well acknowledged by more soil erosion, fewer crop roots, crop residues, and less vegetation cover. Subsequently, soil fertility and organic matters are less accumulated. This precisely shows a high concentration of bulk density (BD), which is an inverse of organic matter. This agrees with Belayneh et al. (2019) and Guadie et al. (2020), who reported that bulk density is an inverse relationship with organic matter. In addition to physical soil property, SWC practices also influenced soil chemical property. A higher pH value was recorded in treated farmland than the untreated one and statistically significant difference between treated and untreated farmland under physical (*p* < 0.01) and integrated (*p* < 0.05) SWC practices, whereas not under biological SWC practices. As a result, the soil acidity of treated farmland is slightly higher (5.79–6.63) than that of untreated farmland (5.37–5.97), which was relatively improved and suitable for crop production. This agrees with Keesstra (2016), who reported that most plant nutrients are available for crop growth at pH values ranging from 5.5 to 7. Tanto and Laekemariam (2019) also confirmed that integrated physical structure with biological measures leads to increase soil pH values on conserved farmland than non-conserved in Southern Ethiopia. The hidden concept behind this point is the availability of organic carbon higher on conserved land than non-conserved land. Besides, why a higher pH value was measured on treated farmland than untreated farmland is that there is less soil loss, high concentrations of basic soil nutrients, organic matter content, and relatively high base saturation percentages (Hailu 2017; Hishe et al. 2017). As reported, soil pH value is higher on land conserved with stone bund and terraced than non-stone bund and non-terraced, respectively. Land, which is rehabilitated with elephant grass and Sesbania, has a higher soil pH value than land degraded via grazing because under elephant grass and Sesbania there is a high concentration of organic matter and basic cations as a result of biomass decomposition (Sinore et al. 2018).

The difference in SOC is statistically significant between treated and untreated farmland under physical (*p* < 0.01) and integrated SWC practices (*p* < 0.01). This suggests that SWC practices played a vital role in reducing soil loss and enhancing the accumulation and retaining of SOM through increasing the decomposing of leaves, roots, and crop residues. This agrees with scholars’ findings from different parts of the country, in which treated farmland has more SOC than untreated farmland (Tanto and Laekemariam 2019; Guadie et al. 2020). Similarly, Demelash and Stahr (2010), and Hishe et al. (2017) also announced that SOC is higher on conserved land than non-conserved. This shows that soil organic carbon is easily taken away by run-off or soil erosion from non-conserved land whereas reverse to non-treated farmland.

The mean value of TN was higher on treated than untreated land use under each intervention. There is strong statistically significant (*p* < 0.01) between treated and untreated farmlands under physical and integrated SWC practices. This implies that physical measures help to curb soil erosion and reduce run-off. This agrees with Hishe et al. (2017); Alemayehu et al. (2020); Bezabih et al. (2016); Guadie et al. (2020), who found that TN is higher on conserved than non-conserved farmland by physical measures. Accumulations of soil organic matter were higher under integrated SWC practices due to the presence of forage or grasses species uses for bund stabilization. This agrees with Tadesse et al. (2016) and Amdemariam et al. (2011), who reported that higher TN value was registered on integrated SWC practices than control. The main reason is that the availability of SOM from various forage species helps for enhancing the TN nutrients available for plant growth.

Available phosphorus has a higher mean value on treated than untreated farmland under three SWC practices and is statistically insignificant (*p* < 0.05). This has a direct relationship with pH and organic carbon results, in which their mean values were higher under-treated farmland. Relatively available phosphorus is higher on farmland treated by integrated than physical and biological treated farmland. This agrees with Tadesse et al. (2016) who reported the mean value of available phosphorus is higher under integrated than non-conserved farmland, and statistically significant. According to their ideas, there is less impact of SWC practice on available phosphorus. Besides, Hailu et al. (2012) and Hishe et al. (2017) reported similar findings. They found that available phosphorus is numerically higher and but statistically significant under a physical measure. However, it disagrees with Bezabih et al. (2016), who reported that available phosphorus is lower on conserved than non-conserved farmland by physical measures. According to Mengistu et al. (2016), the availability of phosphorus concentration varied based on three factors: parent material from soil was formed, the distance between two consecutive structures, and the management level of soil bund.

Treated farmland has a higher SOM value than untreated farmland and is statistically significant (*p* < 0.01) under physical and integrated SWC practices. This agrees with Tadesse et al. (2016), who reported that SOM content is higher on treated farmland under integrated (2.13%) and physical (1.47%) measures than non-conserved (0.85%) farmland. The mean difference value of SOM concentration was recorded increasing order under physical < biological < integrated SWC practices. This shows that the contribution of SWC varied under various soil and water conservation practices. Besides, the availability of crop residues, litterfall, and leaves of forages or shrubs on biological and integrated become decomposed and accumulated SOM nearest to bunds. This agrees with Demelash and Stahr (2010), who ratified that the value of SOM content is higher on treated farmland by integrated than untreated. Therefore, the intervention of SWC practices plays a vital role in combating land degradation, through reducing soil erosion and run-off, increasing the decomposition of various vegetation and crop residues, which in turn rebuilt the accumulation of SOM and the productive capacity of the land.

### Effects of SWC practices on crop yield

Improving crop yield by enhancing the productive capacity of the land is the cornerstone of SWC practices. The synthesis result shows the introduced technology had positive reimbursements regarding crop yield. This is due to the fact reason that relatively most applied practices were improved soil physicochemical properties (Tables 2 and 4). For instance, physical structure (e.g., Fanya juu) increases crop yield up to 204% (Table 5). This is greater than the previous result reported by Ararso et al. (2016). They initiated that crop yield increased by 87% on farmland treated with physical structure (e.g., Fanya juu). However, the amount of yield obtained from treated farmland varied from crop to crop and from place to place. This may happen for two main reasons; one reason is crop variety issues. Different crops grown in the same area brought various amounts of yield, which erupted from the internal components of each crop. The second reason is due to the various characteristics of the study site based on rainfall, altitude, temperature, and soil erosion status, utilization of fertilizer, soil types, and indigenous soil management practice. This agrees with the study conducted by Adgo et al. (2013), Amare et al. (2013), Teshome et al. (2013), Tanto and Laekemariam (2019), whose found that different amount of crop yield was harvested from crop to crop and from place to place from treated farmland in a different site.

Despite a positive contribution of SWC on crop yield, a few structures (e.g., soil bund) can reduce crop yield minimum up to − 6.97% at 5 years and maximum up to − 7.32% at 12 years old (Table 5). This perhaps happened due to various reasons such as physical structure reduces cultivable land from crop to be growing. This coincides with the study reported by Teshome et al. (2013), who found that physical structure reduces cultivable land and crop production. Some soil and water conservations were not constructed according to the guidelines given by FAO (Desta et al. 2005). The study conducted by Wolka et al. (2018) ratified that around 74% of farmers were confirmed that the constructed physical SWC has less quality in Toni and Bokole watersheds of the Omo-Gibe basin. Furthermore, the lack of disintegration of physical with biological measures, less maintenance, and management of implemented structure are also the other factors. This coincides with Bekele et al. (2018) and Wolancho (2015), who reported that some constructed soil and water conservation practices have less quality which was less than the recommended standard. Similar findings were reported by Adimassu et al. (2012) and Sinore et al. (2018), who ratified lack of stabilization of physical structure with biological measure impose crop yield too low. Generally, Fanya juu and stone-faced soil bund increase crop yield consistently as year increases, whereas bund (e.g., soil and stone bund) were inconsistent. Besides, the integrated physical structure with biological measures relatively increases crop yield than physical structure alone. Therefore, to increase the advantages of SWC practices in terms of soil physicochemical property and crop yield, applying the combined physical structure with the biological measure is accountable as an optional strategy.

### Linkage of soil physicochemical properties and crop yield under SWC practices

The mean value of all selected soil physicochemical properties were improved on treated farmland with integrated measures (Amdemariam et al. 2011; Tanto and Laekemariam 2019) and physical structure (Amdemariam et al. 2011; Tanto and Laekemariam 2019; Guadie et al. 2020; Alemayehu et al. 2020; Adimassu et al. 2012) (Tables 2 and 4) and subsequently, crop yield was relatively increased (Amdemariam et al. 2011; Tanto and Laekemariam 2019; Amare et al. 2013; Adgo et al. 2013) (Table 5). The amount of crop yield collected from treated farmland under integrated and physical structures (e.g., Fanya juu and stone-faced soil bund) was consistently higher whereas not under a few physical structures (e.g., soil and stone bunds) as their year increases. Remarkably, SWC practices were relatively returned the productive capacity of land and crop yield. This coincides with the watershed objective, in which enhancing crop yield through restoring soil fertility and reducing soil erosion is the main target of community-based participatory watershed management (Desta et al. 2005). Besides, it relatively minimizes the amount of cost to be paid for buying organic fertilizers. This agrees with Yirga and Hassan (2009), who revealed that farmer’s paid more cost when soil erosion increases.

### Factors affecting crop yield

This study may come with the main finding, factors affecting crop yield, which was overlooked by many scholars. As predicted, age of SWC practices and mean annual rainfall positively and significantly (*p* < 0.01) influenced the amount of crop yield harvested from treated farmland. This implies that as the age of established SWC practices increased by one age (year), it would lead to increases in crop yield by 46.66 coefficients when other variables stay constant. This is because when SWC practice accounted for a long time its contribution in reducing soil erosion, soil loss, and run-off, and inversely it improves some soil physicochemical properties. This agrees with Tanto and Laekemariam (2019), who reported that a selected soil physicochemical properties (pH, SOC, Ava.P, Clay, Silt, BD, and Sand) are improved as a year of applied SWC practices increases. As assumed, mean annual rainfall positively and significantly (*p* < 0.01) influence crop yield obtained from treated farmland. This implies that when mean annual rainfall is relatively increased by one millimeter, the amount of yield that could be obtained from treated farmland increases by 2.91 coefficients when other variables are held constant.

The mean annual temperature negatively but statistically significantly (*p* < 0.01) affected the amount of crop yield obtained from treated farmland which is similar to a predicted. With other variables held constant, when the mean annual temperature is increased by 1 °C, the amount of crop yield decreases by 0.01 coefficient. Finally, mean altitude negatively and significantly (*p* < 0.01) affected the crop yield obtained from treated farmland as predicted. This means that when the mean altitude increases by 1 m above sea level, the amount of yield to be collected will decrease by 0.01 Q/ha at other factors held constant.

## Conclusions

Soil erosion is a critical agenda in the globe including Ethiopia. As a result, it adversely delays the environmental service, crop yield, and the smallholder farmer’s livelihood. This review result shows that soil physicochemical properties were either positively or negatively affected by an intervention of SWC practice. Accordingly, BD was negatively, clay and silt were positively and sand was not affected under a physical measure. Except for clay soil contents, all the selected physical properties were positively affected by biological measures. Similarly, all selected soil chemical properties (SOC, SOM, pH, Ava.P., and TN) were positively affected by physical SWC practices while TN was negatively and positively affected by the biological measure. The mean value of clay and BD had lower under integrated SWC practices. All selected chemical properties had a higher mean value on treated than untreated land under all SWC practices. Crop yield obtained from treated farmland by Fanya juu, stone-faced soil bunds, and integrated physical with biological measure was constantly increasing as year increases while inconsistent under some physical measure (e.g., stone and soil bund). The regression model reported that year (age) of implemented SWC practices and mean annual rainfall positively and significantly influenced crop yield obtained from treated farmland whereas mean annual temperature and attitude did negatively. Generally, SWC practices play a great role in modifying soil physicochemical properties and crop yield. Thus, it should be encouraged by reducing its negative effects and increasing its positive by implementing a technology following a guideline, integrating physical measure with biological measure, and increasing its management. This should not only the task of smallholder farmers but also it needs due attention from the government and any concerned body. Also important to mention is we lack strong scientific evidence that can testify the dependable impact of Ethiopian government efforts of land rehabilitation campaign for a couple of decades, which will attract upcoming researchers.

## Data Availability

Not applicable.

## Appendix

See Table S1.

**Table S1:** Detailed description of a developed criterion for inclusion and exclusion of literature

Criteria	Categories	
	Inclusion	Exclusion
Study area	Four regional states (Oromia, Amhara, SNNP, and Tigray) (Fig. 1)	All regional states except four regions
Characteristics of the study area	Mean annual temperature, mean annual rainfall, mean altitude, SWC practices, and crop cultivation	All except the selected characteristics
Physical structure	Soil bund, Fanya juu, Stone bund, and stone-faced soil bund, let it (A)	All physical structures except the selected one (A)
Biological measure	Forage (e.g., grasses or shrubs) used as a hedgerow or grass strip, let it (B)	All biological measures used as SWC practices except for the selected (B)
Integrated SWC practices (physical + biological)	A + B means that biological SWC practices which are used as physical structure stabilizer, let it (C)	All expect the combination of A + B SWC practices
Land use	Farmland, which is treated with either by A, B, or C and untreated farmland adjacently	All land-use types except farmland treated with either A, B, or C and its untreated farmland adjacently
Year of publication	Literature which was published between 2010 up to 2020, about the effects of A, B, and C SWC practices on selected (D and E) soil physicochemical properties and crop yield	All published literature before 2010
Soil chemical property	Available phosphorus (Ava. P), Total nitrogen (TN), soil reaction (pH), soil organic matter (SOM), and soil organic carbon (SOC), let it (D)	All soil chemical properties except the selected one (D)
Soil physical property	Bunk density (BD) Silt, sand, and clay, let it (E)	All soil physical properties except the selected one (E)
Crop yield	All cereal crop yields collected from treated farmland with A, B, and C and untreated land adjacently	All crops except the included one
Year (age) of SWC practices	This is based on the time interval between the implementation of SWC practices up to evaluation. Thus, the year of SWC practices must be greater than or equal to 2 years	Year of SWC practice for less than 2 years

## Abbreviations

NGOs: Non-Governmental Organizations
SWC: Soil and water conservation
SNNP: Southern Nations, Nationalities and Peoples
SPSS: Statistical Packages for Social Scientists
